# Influence of Particle Size Distribution on Rheology Behavior of Slurry and Flow Characteristics of Long-Distance Transportation

**DOI:** 10.3390/ma19091881

**Published:** 2026-05-02

**Authors:** Xin Chen, Zhongtao Jiang, Junhui Zhang, Zeyu Li

**Affiliations:** 1 School of Resources and Safety Engineering, Central South University, Changsha 410083, China; chenxin_ck@csu.edu.cn (X.C.);; 2 Sinosteel Maanshan General Institute of Mining Research Co., Ltd., Ma’anshan 243000, China; 3 School of Resources and Safety Engineering, University of Science and Technology Beijing, Beijing 100083, China; 4 School of Geology and Mining Engineering, Xinjiang University, Urumqi 830046, China; 5 Collaborative Innovation Center of Green Mining and Ecological Restoration for Xinjiang Mineral Resources, Urumqi 830046, China

**Keywords:** particle size distribution, cement paste backfill, rheology behavior, long distance, characteristics of flow

## Abstract

**Highlights:**

- The relationship between mixed aggregate particle size distribution and slurry rheological parameters was established.
- Tailings particle size distribution significantly affects slurry transport resistance, whereas flow velocity is relatively insensitive to particle size distribution.
- Particle segregation occurs under long-distance transport conditions, and particle size distribution significantly influences particle distribution.

**Abstract:**

The particle size distribution of backfill aggregate is a key factor affecting the performance of the -long-distance pipeline transport of backfill slurry. However, the understanding of its impact on slurry flow behavior, transportation resistance, and particle distribution mechanisms remains incomplete and calls for further investigation. This study first obtained the rheological parameters of slurry and their variation laws under the influence of particle size distribution through rheological experiments. Subsequently, CFD numerical simulations are used to investigate the flow characteristics of slurry under long-distance transportation conditions. The findings demonstrate that a reduction in the mixed aggregate particle size leads to a significant increase in both the yield stress and plastic viscosity of the backfill slurry. The conveying distance shows a positive correlation with the slurry transportation resistance. Furthermore, the slurry exhibits plug flow behavior in both the horizontal and vertical pipe sections, whereas this plug flow pattern is no longer observed in the bend section. The tailings particles exhibit a distinct stratified distribution within the pipeline. In the horizontal pipe section, the graded tailings predominantly settle at the bottom, whereas the fine tailings remain suspended near the top. In contrast, in the vertical pipe section, the graded tailings tend to accumulate in the central zone of the pipe, while the fine tailings are dispersed along the pipe wall. As the content of graded tailings increases from 30% to 50%, both the zones with increased and decreased particle volume fractions expand, while the steady flow zone correspondingly shrinks. Meanwhile, the volume fraction of graded tailings at the bottom of the pipe rises significantly from 0.12 to 0.61. This research provides important theoretical support for the optimized matching and rational application of tailings particle size distribution in the design of long-distance pipeline transportation systems for mine backfill.

## 1. Introduction

With the rapid advancement of industrial development, the demand for non-ferrous metal resources has been increasing year by year. However, shallow mineral resources can no longer satisfy this growing demand, rendering deep mining an inevitable necessity [1]. With the continuous increase in mining depth, both the layout length and vertical elevation of backfill pipelines have progressively expanded. As a result, the pipeline transport of backfill slurry encounters significant challenges, including long transportation distance, large vertical drop, high stowing gradient, and high transportation resistance [2,3,4,5]. Concurrently, within the background of zero tailings mining, the incorporation of −20 μm fine tailings into coarse tailings as mixed aggregates for backfill slurry preparation has emerged as an effective strategy for tailings reutilization. However, during actual pipeline transportation, the backfill slurry is prone to issues such as pipe blockage and pipe bursting. These problems become more frequent and severe in long-distance conveying, significantly constraining the efficiency and safety of backfill mining operations [6,7,8,9,10,11,12,13]. Fundamentally, these issues arise from a lack of in depth understanding of the slurry rheology behavior and long-distance pipeline transport characteristics under the influence of tailings particle size distribution.

A precise understanding of the influence of tailings particle size distribution on the rheological behavior of backfill slurry is essential for guiding the design of backfill pipeline systems. Zhang et al. [14] explored the relationship between the rheological parameters of CPB slurry and the content of graded tailings through rheological tests, and found that the yield stress and plastic viscosity of CPB slurry were negatively correlated with the content of graded tailings. Cheng et al. [15] studied the influence of tailings particle size distribution on the yield stress of CPB slurry, and the results showed that the yield stress increased with the increase in fine particle content. Zhang et al. [16] investigated the rheological parameters of CPB slurry prepared by mixing overflow tailings and graded tailings, and the findings demonstrated that the increase in fine tailings content enhanced the yield stress and plastic viscosity. Kaushal et al. [17] tested the pressure drop of particles with two particle sizes (440 μm and 125 μm) using a loop pipe system, and the results showed that fine particles had greater friction loss than coarse particles during high concentration slurry transportation. Research on the influence of particle size distribution on the rheological behavior of backfilling slurry has become a prominent yet challenging topic. However, its effects on slurry flow characteristics have not yielded universally applicable conclusions, making it difficult to directly apply existing findings to engineering practice. This limitation may be attributed to the uniqueness and variability of tailings properties arising from different production processes. Therefore, it is necessary to conduct targeted investigations on the pipeline transportation performance of backfill slurry prepared from specific tailings.

The transportation performance of backfill slurry is commonly assessed through laboratory L-pipe tests and, in some cases, full loop-pipe experiments, both of which are time-consuming and material-intensive. By comparison, computational fluid dynamics (CFD) simulations have increasingly become an efficient and reliable tool for investigating the flow characteristics of backfill slurry during pipeline transportation [18,19,20,21,22,23,24,25]. Dong et al. [26] used the CFD mixture model to study the transportation state of CPB slurry containing coarse particles at elbows, and the results showed that the distribution of coarse particles was related to the pipe type, and their position near the pipe wall was affected by the velocity magnitude. Meanwhile, the velocity of CPB slurry increased at elbows, and the high-velocity zone gradually moved to the outer wall of the elbows. Wu et al. [27] simulated the long-distance pipeline transportation performance of high-viscosity slurry via CFD, and the results indicated that the transportation resistance of high-mud full tailings paste without a pumping agent was 2 to 4 times higher than that with a pumping agent. Liu et al. [28] used the CFD method to study the pipeline transportation characteristics of CPB slurry considering cement hydration. The results showed that during pipeline transportation, coarse particles were deposited at the bottom of the pipeline, while fine particles were suspended at the top. In the aforementioned studies, numerical simulations have typically employed simplified geometric models, such as L-pipe, bends, or horizontal pipe sections, with model lengths generally limited to approximately 10 m. These configurations do not correspond to the actual pipeline lengths used in engineering practices, thereby limiting the applicability of the results to long-distance transportation scenarios, and the influence of particle size distribution on transport characteristics over conveying distances of several kilometers cannot be adequately captured.

In summary, under long-distance pipeline transportation conditions extending over several kilometers, studies addressing the influence of tailings particle size distribution on the transport performance of backfill slurry remain limited. Therefore, it is essential to investigate this issue through an integrated approach combining laboratory experiments and numerical simulations. Accordingly, this study intends to first test the rheological parameters of the slurry and elucidate their relationship with particle size distribution through rheological testing. Subsequently, the effects of particle size distribution on slurry transportation resistance, velocity distribution, and volume fraction distribution during long-distance pipeline conveying will be systematically evaluated.

## 2. Materials and Methods

### 2.1. Backfill System Overview

**Figure 1:**
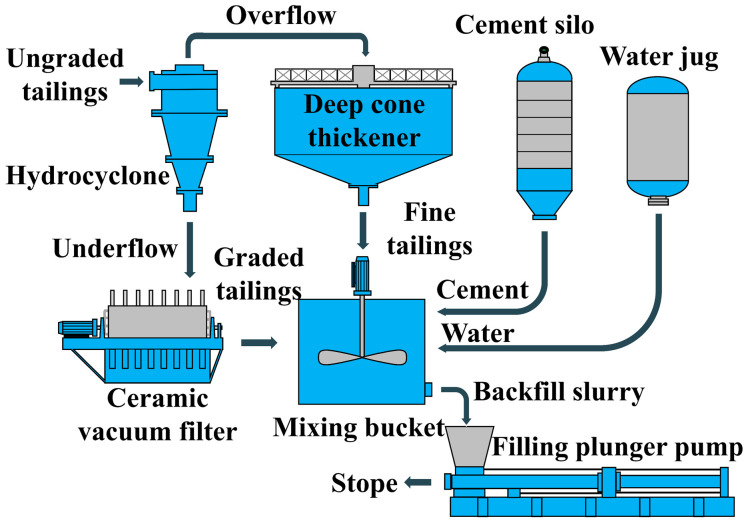
Tailings treatment process..

Figure 1 shows the tailings treatment system and backfill slurry preparation system of Fankou Lead-Zinc Mine. The raw tailings slurry generated from the concentrator is transported to a hydrocyclone for the screening of coarse and fine particles. Tailings particles with a size of +20 μm are discharged from the underflow port of the hydrocyclone and further dewatered by a ceramic vacuum filter press to make the tailings concentration reach more than 85%. The dewatered tailings are graded tailings (GT). In contrast, tailings particles with a size of −20 μm are discharged from the overflow port of the hydrocyclone, then enter a deep cone thickener and are concentrated into tailings slurry with a concentration of more than 55% under the action of gravity before being discharged from the underflow port, the discharged tailings slurry is fine tailings (FT). At the start of backfill operations, the treated GT and FT are uniformly mixed with cement and water in a mixing bucket to prepare backfill slurry, which is then pumped to the stope requiring backfill operations by a backfill plunger pump.

### 2.2. Materials

**Figure 2:**
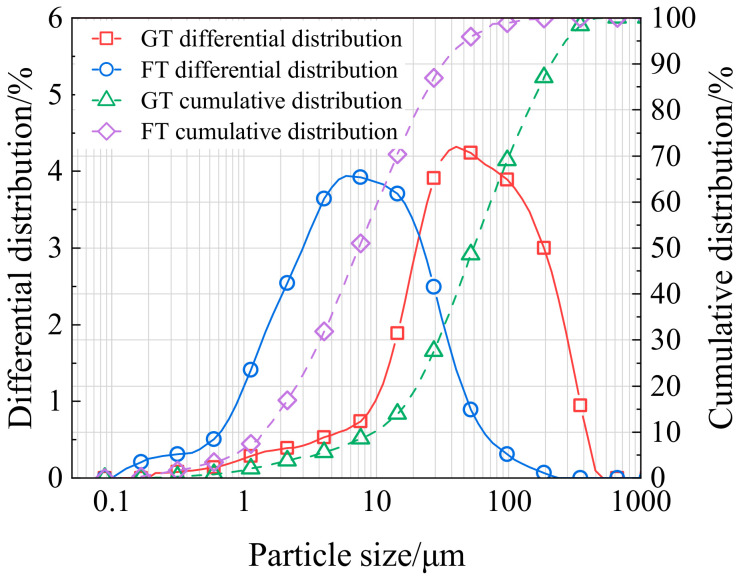
Particle size distribution of GT and FT.

The basic physical properties of the tailings used in the experiments are presented in Table 1 and Figure 2. The average particle size of GT is 80.35 μm, with particles smaller than 20 μm accounting for 20.85%. In contrast, FT has an average particle size of 12.74 μm, with 81.03% of the particles smaller than 20 μm. The uniformity coefficient (Cu) and curvature coefficient (Cc) were used to evaluate the gradation uniformity of the two types of tailings [29]. The Cu and Cc of GT are 7.90 and 1.32, respectively, while those of FT are 8.03 and 1.04, respectively, indicating that both GT and FT are well graded with continuous gradation curves. Moreover, FT exhibits slightly better gradation characteristics and a more continuous gradation curve compared to GT. The binder used in the experiments was PO 42.5 Portland cement, and ordinary tap water was used for slurry preparation.

### 2.3. Research Scheme Design

In the design of recipe of cement paste backfill (CPB) slurry, the flow performance of slurry is one of the primary considerations. It is generally believed that the content of −20 μm particles in tailings should not be less than 15% to ensure the flowability of the slurry in the pipeline [30]. In this study, the GT to FT ratio was set in the range of 30%:70% to 50%:50%. After mixing GT and FT, the proportion of particles smaller than 20 μm ranged from 51% to 63%, which satisfies the requirement that the −20 μm content should not be lower than 15%, while maximizing the recovery and utilization of tailings. Meanwhile, the mass concentration of the slurry is set to 66%, 67%, and 68%, and the specific test scheme is presented in Table 2.

To better describe the proportional relationship between GT and FT, the mixed particle size (*MPS*) is used to describe the particle size of the tailings mixed with GT and FT, which can be calculated by the following formula:

(1) MPS=GTC⋅GTPS+FTC⋅FTPS

where *MPS* is the mixed tailings particle size, μm; *GTC* and *FTC* are the contents of GT and FT, wt%; *GTPS* and *FTPS* are the particle sizes of GT and FT, respectively, which are replaced by the median particle size (*d*_50_).

### 2.4. Slurry Rheology Test Methods

The Anton paar MCR72 rheometer (Aton Paar(Shanghai) Trading Co., Ltd., Shanghai, China) was used to test the rheological properties of the backfill slurry. The instrument has a rotational speed range of 0.001 rpm to 1500 rpm, an angular frequency range of 0.001 rad/s to 628 rad/s, a maximum torque of 125 mNm, and a torque resolution of 100 nNm. The vane rotor, with minimal disturbance during slurry insertion, effectively prevents wall slip effects and is widely used in the rheological testing of backfill slurry. In this study, a vane rotor with a diameter of 20 mm and height of 40 mm was employed, and the testing gap size was set to 20 mm to minimize wall slip and boundary effects on the results.

During the pipeline transport of backfill slurry, the shear rate distribution within the pipe is uneven, with a maximum typically occurring near the pipe wall. Based on the conditions set in the numerical simulation, the maximum shear rate within the pipe is found to be 200 s^−1^. Since backfill slurry behaves as a typical Bingham fluid, its apparent viscosity stabilizes as the shear rate increases. Therefore, the apparent viscosity for a wider shear rate range can be determined through testing within a specific shear rate range. In this study, the slurry flow behavior within the shear rate range of 0 to 200 s^−1^ in the pipeline is represented by a specific shear rate range of 0 to 70 s^−1^. The variable shear rate mode was adopted, with a shear rate range of 0 to 70 s^−1^, a shear rate change rate of 0.7 s^−1^, and a shearing time of 90 s. The data points collected during the test were used to draw rheological curves, based on which the rheology behavior of the slurry was analyzed [12].

**Figure 3:**
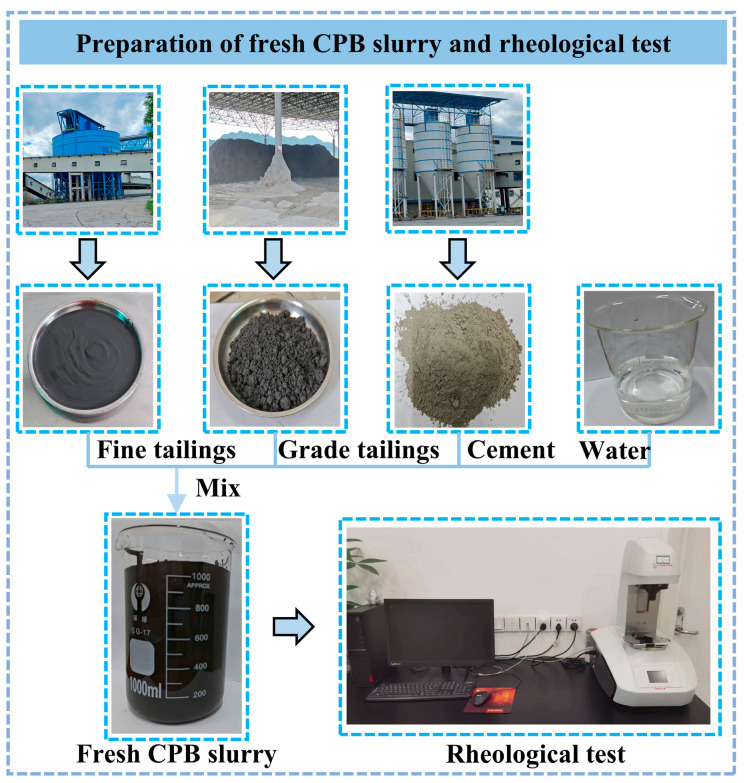
Fresh CPB slurry rheological test.

Considering the high concentration of GT in the slurry, rheological testing was conducted immediately after slurry preparation to minimize the effect of tailings settlement on the results. The entire rheological testing process was completed within 5 min, with the test environment maintained at a temperature of 20 ± 2 °C. Each group of rheological tests was repeated 3 times to ensure the reliability of the measurement results. The testing procedure is shown in Figure 3.

### 2.5. CFD Simulation of Long-Distance Pipeline Transportation

#### 2.5.1. Physical Model

**Figure 4:**
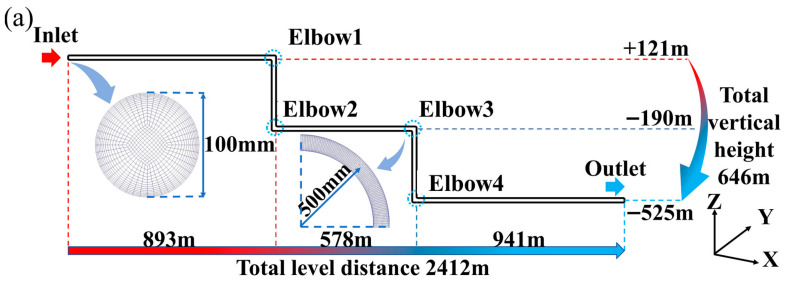

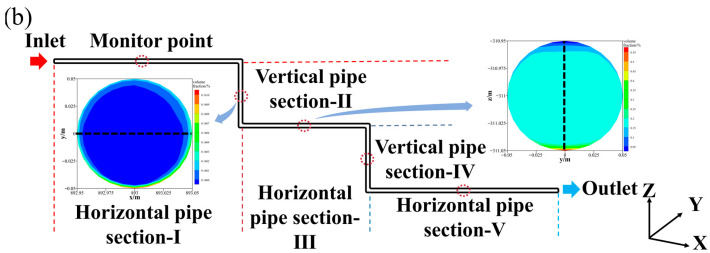
Backfill pipeline diagram (**a**) geometric model; (**b**) monitor point.

Based on the actual underground pipeline layout of the mine, Ansys SCDM 2019R3 was used to construct a 3D physical model of the pipeline system, and the specific structure of the model is shown in Figure 4a. The core dimensions of the pipeline physical model are designed as follows: the pipeline consists of horizontal and vertical sections, where the horizontal section is 2412 m in length, the vertical section is 646 m in height, and the total length of the pipeline reaches 3058 m. The inner diameter of the pipeline is 100 mm, and the outer diameter is 120 mm. In the pipeline model, the horizontal pipeline and vertical pipeline are connected by a 90 ° elbow with a radius of 500 mm. In Ansys ICEM 2019R3, the mesh was generated using a sweep meshing approach. Local mesh refinement was applied to the pipe sidewalls and the elbow region. The total number of elements was 2,690,787. A mesh independence study was conducted, and the results confirmed that the simulation outcomes obtained with this mesh resolution are reliable.

To accurately describe the flow characteristics of the slurry at different positions in the pipeline, the pipeline was divided into horizontal and vertical sections. The horizontal section includes three parts, namely I, III, and V; the vertical section includes two parts, namely II and IV. Monitoring points were established at the midpoints of each segment to extract local slurry velocity and volume fraction data.

#### 2.5.2. Mixture Model

The backfill slurry is a high concentration solid–liquid mixture, and the mixture model in the multiphase flow model was adopted to describe the solid–liquid phase behavior [31,32].

The continuity equation of the mixture is expressed as:

(2) $$\frac{\partial }{\partial t}\left(\rho_{m}\right)+\nabla \cdot (\rho_{m}{\vec{v}}_{m})=0$$

${\vec{v}}_{m}$ denotes the mass-averaged velocity, which is expressed as:

(3) $${\vec{v}}_{m}=\frac{\sum_{k=1}^{n}\alpha_{k}\rho_{k}{\vec{v}}_{m}}{\rho_{m}}$$

*α_k_* denotes the volume fraction of phase *k*. In this study, water was regarded as the primary phase, GT as the second phase, FT as the third phase, and cement as the fourth phase. *ρ_m_* denotes the density of the mixture, which is expressed as:

(4) $$\rho_{m}=\sum_{k=1}^{n}\alpha_{k}\rho_{k}$$

The momentum equation of the mixture can be obtained by summing the individual momentum equations of each phase, which can be expressed as:

(5) $$\frac{\partial }{\partial t}(\rho_{m}\vec{v_{m}})+\nabla \cdot (\rho_{m}\vec{v_{m}}\vec{v_{m}})=-\nabla p+\nabla \cdot u_{m}\left[\left(\nabla \vec{v_{m}}+\nabla \vec{v_{m}^{T}}\right)-\frac{2}{3}\nabla \cdot \vec{v_{m}}I\right]+\rho m\vec{g}+\vec{F}-\nabla \cdot \left(\sum_{k=1}^{n}\alpha_{k}\rho_{k}{\vec{v}}_{dr,k}{\vec{v}}_{dr,k}\right)$$

where *n* denotes the number of phases, $\vec{F}$ is the body force, and *u_m_* is the viscosity of the mixture, which can be written as:

(6) $$u_{m}=\sum_{k=1}^{n}\alpha_{k}u_{k}$$

${\vec{v}}_{dr,k}$ denotes the drift velocity of the secondary phase *k*, which can be written as:

(7) $${\vec{v}}_{dr,k}={\vec{v}}_{k}-{\vec{v}}_{m}$$

To further understand the particle distribution of GT and FT during transportation, it is necessary to solve the volume fraction of the secondary phase *k*. The volume fraction equation of the secondary phase *k* can be derived from the continuity equation of the secondary phase *k*, which is expressed as:

(8) $$\frac{\partial }{\partial t}\left(\alpha_{k}\rho_{k}\right)+\nabla \cdot \left(\alpha_{k}\rho_{k}{\vec{v}}_{m}\right)=-\nabla \cdot \left(\alpha_{k}\rho_{k}{\vec{v}}_{dr,k}\right)$$

#### 2.5.3. Boundary Condition

The reliability of numerical simulation results in guiding engineering practice depends strongly on the reasonable setting of boundary conditions. In this study, the boundaries of the fluid domain were set as follows: the inlet was set as velocity-inlet with a velocity of 2.5 m/s, the outlet was set as outflow. Due to the high content of fine particles in the backfill slurry, slip motion between the fluid and the pipe wall occurs. However, this study focuses on the flow characteristics of the slurry under different aggregate particle size distribution, and the impact of wall slip on flow behavior is not considered. The pipe wall is assumed to be a no-slip condition [24,25,26]. Additionally, to accurately capture the sedimentation behavior of coarse and fine particles during pipeline transport under gravitational effects, the gravitational acceleration in the model was set to −9.8 m/s^2^.

Determining the flow regime of the slurry within the pipeline is essential for selecting the appropriate governing equations. The flow regime is typically evaluated using the Reynolds number. When R_e_ exceeds 2300, the flow is considered turbulent; otherwise, it is laminar. The Reynolds number is calculated as follows:

(9) $$R_{e}=\frac{\rho vD}{\mu }$$

where *R_e_* is the Reynolds number; *ρ* is the slurry density; *v* is the flow velocity; *D* is the pipe diameter and *μ* is the viscosity of the slurry.

Based on the rheological parameters and flow velocity, the Reynolds number is less than 2300, indicating that the slurry exhibits laminar flow within the pipeline.

## 3. Results and Discussion

### 3.1. Rheology of CPB Slurry

**Figure 5:**
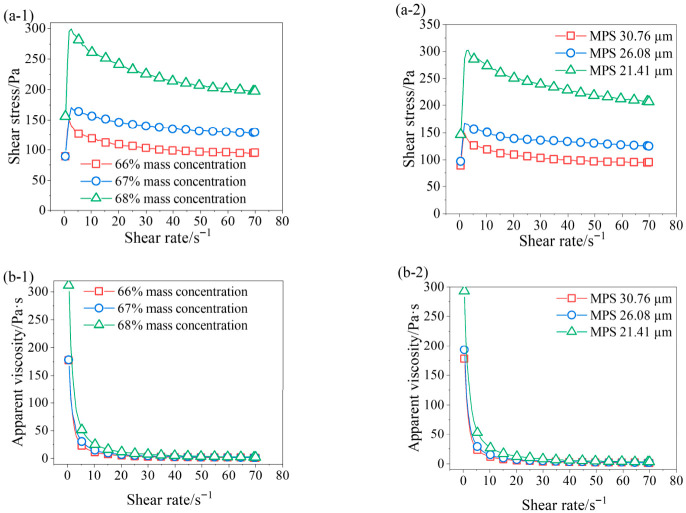
Variation in shear stress with shear rate (**a-1**) different mass concentration; (**a-2**) different particle size distribution; variation in apparent viscosity with shear rate (**b-1**) different mass concentration; (**b-2**) different particle size distribution.

Figure 5(a-1,a-2) shows the relationship between shear stress and the shear rate of fresh backfill slurry under different mass concentrations and MPS, respectively. When the shear rate is below 1.9 s^−1^, the shear stress gradually increases with the increase in shear rate and reaches a peak. This is attributed to the inability of the internal structure of the slurry to respond promptly to the shear rate at low shear rate ranges, resulting in a stress overshoot phenomenon. As the shear rate further increases from 1.9 s^−1^ to 70 s^−1^, the shear stress progressively decreases with increasing shear rate. Due to the thixotropy of the backfill slurry, the rate of structural breakdown exceeds that of structural rebuilding under shear, resulting in a reduction in shear stress and apparent viscosity. The results in Figure 5(a-1) indicate that at a given shear rate, an increase in mass concentration leads to a higher shear stress. Specifically, at a shear rate of 70 s^−1^, increasing the mass concentration from 66% to 68% results in an increase in shear stress from 95.4 Pa to 197.7 Pa. The results in Figure 5(a-2) show that at the same shear rate, decreasing the MPS leads to an increase in shear stress. When the shear rate is 70 s^−1^, the shear stress increases from 95.4 Pa to 206.8 Pa as the MPS decrease from 30.76 μm to 21.41 μm.

Apparent viscosity is defined as the ratio of shear stress to shear rate for a non-Newtonian fluid at a specific shear rate, reflecting the intensity of internal friction within the fluid. Figure 5(b-1,b-2) shows the relationship between the apparent viscosity and shear rate of backfill slurry under different mass concentrations and MPS. The results indicate that the backfill slurry exhibits obvious pseudoplastic behavior. As the shear rate increases, the apparent viscosity gradually decreases and eventually approaches a stable value. Overall, the apparent viscosity increases with the increase in mass concentration and decrease in MPS, and the differences gradually narrow with the increase in shear rate.

Rheological models can effectively describe the flow behavior of CPB slurry, and the Bingham model and Herschel–Bulkley model are commonly used [33,34,35]. The Bingham model is expressed as:

(10) $$\tau =\tau_{0}+\eta \cdot \overset{\cdot }{\gamma }$$

where *τ*_0_ is the yield stress, Pa; *η*_0_ is the plastic viscosity, representing the flow resistance of the fluid once the yield stress has been exceeded, Pa·s; $\overset{\cdot }{\gamma }$ is the shear rate, s^−1^.

The Herschel–Bulkley model is expressed as:

(11) $$\tau =\tau_{0}+K\cdot {\overset{\cdot }{\gamma }}^{n}$$

where *τ*_0_ is the yield stress, Pa; *K* is the consistency coefficient, Pa·s^n^; $\overset{\cdot }{\gamma }$ is the shear rate, s^−1^; n is the flow index, 1.

Table 3 presents the rheological parameters obtained from fitting using two rheological models. The R^2^ for the Bingham model ranges from 0.84 to 0.97, while that for the Herschel–Bulkley model ranges from 0.58 to 0.97. These results indicate that the Bingham model provides a more suitable description of the rheological behavior of the slurry in this study.

**Figure 6:**
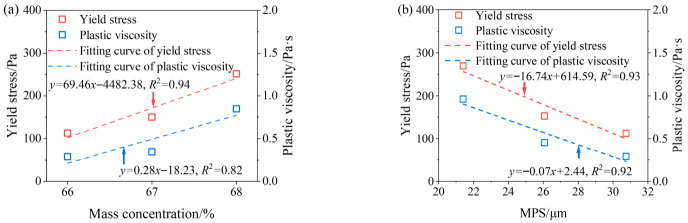
Variation in rheological parameter (**a**) mass concentration; (**b**) MPS.

Figure 6a shows the relationship between slurry mass concentration and rheological parameters. With the increase in slurry mass concentration, both yield stress and plastic viscosity increase. When the slurry mass concentration increases from 66% to 68%, the yield stress increases from 112.4 Pa to 251.3 Pa, and the plastic viscosity increases from 0.29 Pa·s to 0.85 Pa·s. As the mass concentration of CPB slurry increases, the thickness of water film wrapping the particle surface decreases, the particle spacing shortens, and the mutual friction increases. This leads to an increase in the shear stress required to break the internal structure of the CPB slurry during shearing, thereby increasing the yield stress. In addition, the flow of CPB slurry needs to overcome greater friction during shearing, resulting in a corresponding increase in plastic viscosity.

Figure 6b shows the relationship between MPS and rheological parameters. It can be seen that MPS has a significant impact on the rheological parameters of the slurry, larger MPS leads to lower yield stress and plastic viscosity. When the MPS increases from 21.41 μm to 30.76 μm, the yield stress decreases from 268.9 Pa to 112.4 Pa, and the plastic viscosity decreases from 0.96 Pa·s to 0.29 Pa·s. As the tailings particle size decreases, larger flocculated structures are formed between particles, accompanied by an increased frequency of particle collisions. Meanwhile, the specific surface area of the tailings increases, leading to higher water demand and the formation of stronger floc structures. Consequently, greater shear stress is required to break these flocs during shearing, resulting in an increase in yield stress. In addition, the flow of CPB slurry needs to overcome greater internal friction, thereby increasing the plastic viscosity [16].

As shown in Figure 6a,b, the fitting curves exhibit R^2^ values ranging from 0.82 to 0.94, indicating a strong linear correlation.

Accurate selection of appropriate rheological parameters is fundamental for characterizing slurry flow behavior in pipelines. In practical engineering applications, mine designers are primarily concerned with the minimum stress required to initiate flow from a static state, namely the static yield stress of the slurry. Meanwhile, due to the velocity gradient within the pipeline, the shear rate distribution is non-uniform, and the apparent viscosity is therefore required to characterize the interlayer flow resistance of the slurry. Accordingly, in this study, the static yield stress and apparent viscosity are adopted to describe the rheological behavior of the slurry during pipeline transport.

### 3.2. Long-Distance Pipeline Flow Characteristics

#### 3.2.1. Characteristics of Pressure

**Figure 7:**
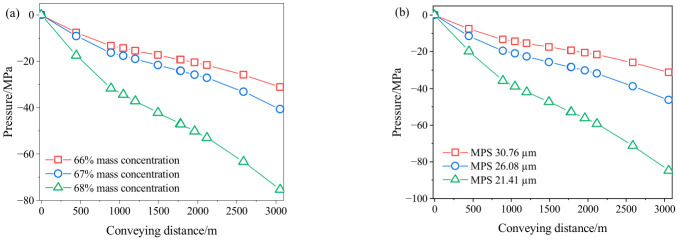
Variation in pressure with conveying distance (**a**) different mass concentration; (**b**) different MPS.

The total pressure at the pipe inlet is defined as zero, and the transport resistance is calculated as the difference between the total pressure at different positions along the pipeline and that at the inlet. The relationship between transport resistance and conveying distance is presented in Figure 7. The pipeline transportation resistance of the slurry gradually increases with the increase in transportation distance. The increase in transportation distance leads to higher energy consumption during transportation, thereby increasing the pipeline transportation resistance.

Figure 7a shows that at the same distance, transportation resistance increases with mass concentration. When the transportation distance is 3058 m, the resistance increases from 31.2 MPa to 75.3 MPa as the slurry mass concentration increases from 66% to 68%. The results in Figure 7b show that at the same transportation distance, the resistance gradually increases with the decrease in MPS, and the increasing amplitude of resistance gradually increases. At 3058 m, resistance increases by 48.1% as MPS decreases from 30.76 μm to 26.08 μm, and then by 83.3% as MPS decreases to 21.41 μm.

CPB slurry is a typical Bingham fluid and must overcome its yield stress in order to flow within the backfill pipeline. The higher the yield stress, the greater the energy required to initiate flow, leading to increased energy consumption during transportation. Meanwhile, a higher plastic viscosity intensifies the friction between solid particles and the pipe wall as well as among the particles themselves, thereby increasing flow resistance and the energy required to overcome it [36]. Therefore, an increase in mass concentration and a decrease in MPS results in higher yield stress and plastic viscosity, which in turn leads to increased slurry transportation resistance.

The pipeline transportation resistance of backfill slurry is typically overcome by the combined effects of gravitational potential energy and pumping pressure. When the transportation resistance becomes excessively high, it not only significantly increases the energy consumption of the pumping system but also imposes higher requirements on the power matching and operational stability of the pumping equipment, potentially compromising system safety. Therefore, in engineering practice, it is necessary to reasonably control the slurry mass concentration and the particle size distribution of the backfill aggregates according to the conveying distance, ensuring they remain within an appropriate range so as to guarantee good fluidity and the stable pipeline transportation performance of the backfill slurry.

The pipeline transport resistance of slurry is influenced not only by external conditions but also by its intrinsic properties, such as thixotropy and wall slip effects, which were not considered in the present study. Previous studies have reported that, due to thixotropy, the transport resistance decreases significantly with increasing transport time and eventually approaches a stable value [37,38]. Under long-distance pipeline transport conditions, the slurry experiences prolonged flow, and the time required to reach a steady-state flow is relatively short. As a result, the thixotropic behavior becomes more pronounced, leading to a more significant reduction in transport resistance. In this study, the numerical simulations are conducted under steady-state flow conditions, which better represent the flow state of the slurry after thixotropic structural evolution. In addition, Xue et al. [39] reported that shear-induced particle migration causes particles to move from the pipe wall toward the pipe center, resulting in the formation of a slip layer near the wall. The presence of this slip layer reduces the effective transport resistance compared to conditions where wall slip is neglected. This effect becomes increasingly significant under long-distance pipeline transport conditions.

#### 3.2.2. Characteristics of Velocity

**Figure 8:**
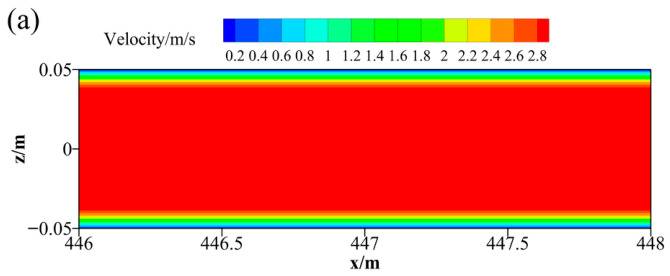

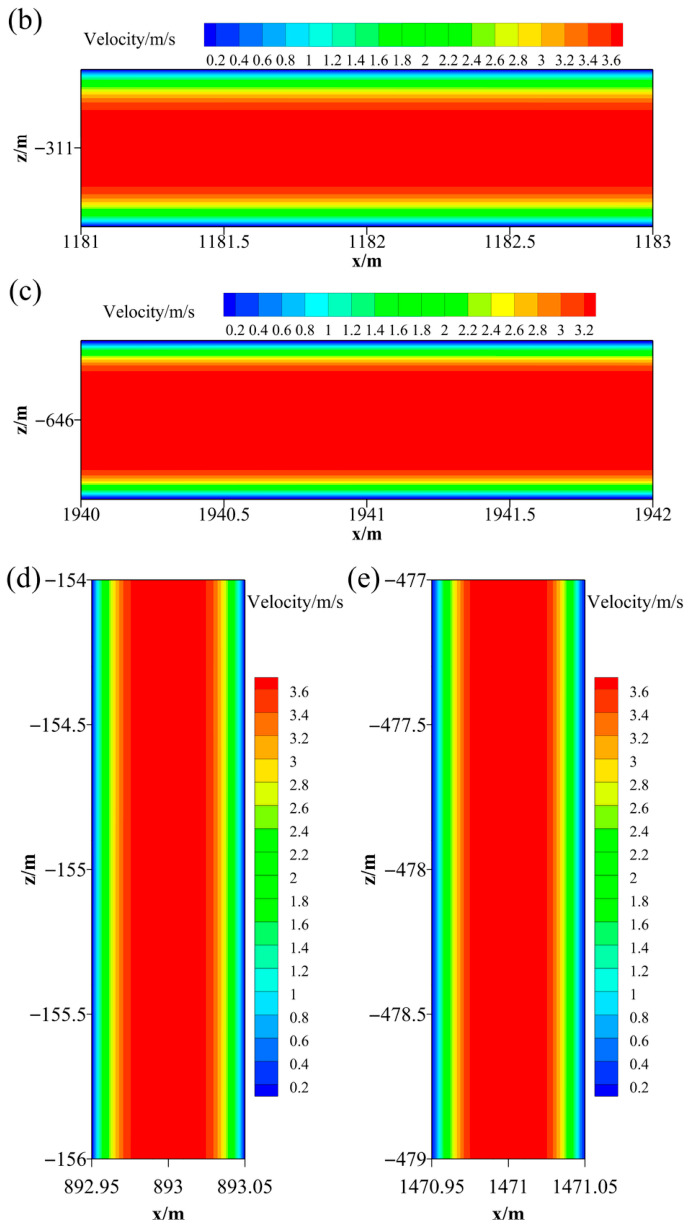
Velocity distribution of CPB slurry (**a**) horizontal pipe section-I; (**b**) horizontal pipe section-III; (**c**) horizontal pipe section-V; (**d**) vertical pipe section-II; (**e**) vertical pipe section-IV.

Figure 8 presents the velocity contours of the slurry at monitoring points in different pipe sections. The flow in the selected sections is fully developed. It can be observed that the slurry exhibits plug flow behavior in both the horizontal and vertical pipe sections. The central region forms the plug flow zone, where the maximum velocity reaches 3.7 m/s and remains relatively stable. The surrounding region is characterized by shear flow, in which the velocity gradually decreases with a certain gradient from the pipe center toward the pipe wall, eventually approaching a stagnant state, indicating that the slurry flows in a laminar regime within the pipeline.

**Figure 9:**
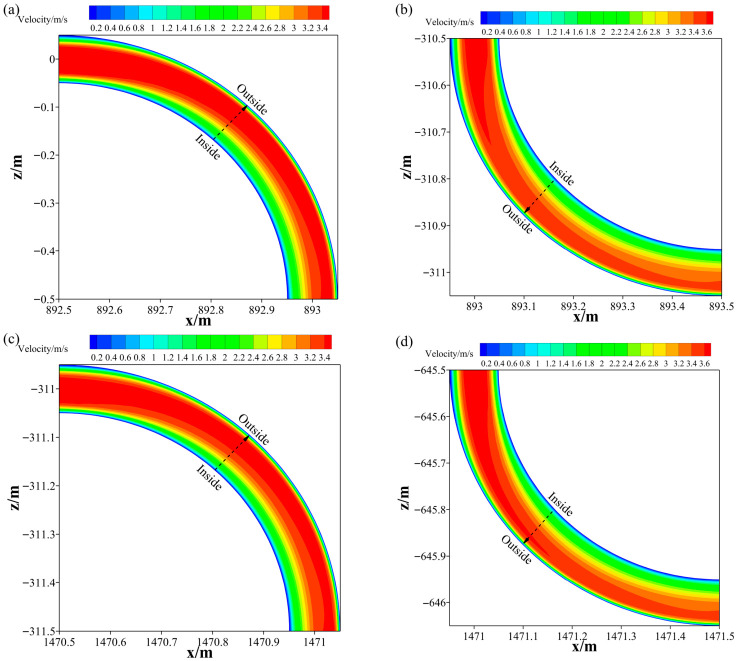
Velocity distribution of CPB slurry in bent pipe section (**a**) elbow 1; (**b**) elbow 2; (**c**) elbow 3; (**d**) elbow 4.

Figure 9 shows the velocity contours of the slurry at each elbow. Slurry flow at elbows differs from that in horizontal and vertical sections and no longer forms plug flow. Velocity increases from the inside to the outside of the pipeline with a gradually increasing gradient; the high-velocity zone is concentrated on the outside, and the low-velocity zone on the inside.

**Figure 10:**
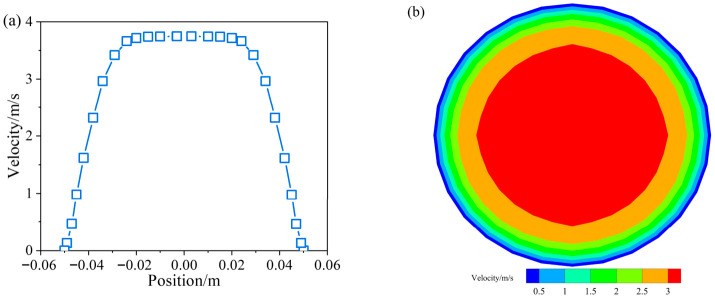
Velocity distribution at the outlet section (**a**) different radial points; (**b**) cross-section.

Figure 10a shows the relationship between the slurry velocity and position at the pipeline outlet. Velocity increases rapidly from 0 to 3.7 m/s from the wall to the center and then remains constant. Figure 10b presents the velocity contour of the slurry at the cross-section of the outlet, from which it can be seen that the velocity distribution is concentric circular, with a significant velocity gradient.

**Figure 11:**
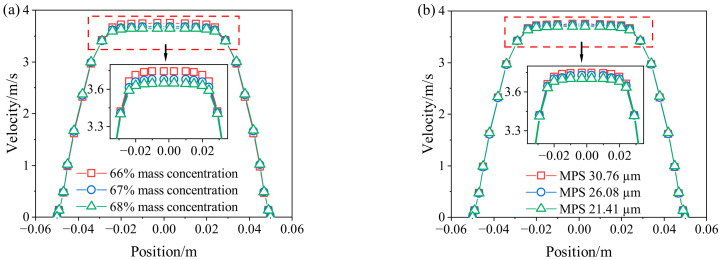
CPB slurry velocity at different radial points of the outlet section (**a**) different mass concentration; (**b**) different MPS.

The velocity data at the pipeline outlet in the z range of −646.05 m to −645.95 m were extracted, and the results are presented in Figure 11. It can be observed that the velocity across the pipe cross-section is relatively insensitive to variations in concentration and MPS.

In the numerical simulations, a no-slip boundary condition was assumed at the pipe wall, resulting in a velocity of zero near the wall. However, in actual pipeline transport, the slurry is subjected to continuous shear, which induces particle migration from the pipe wall toward the pipe center. This process leads to the formation of a thin lubrication layer, primarily composed of water, near the wall, causing the velocity in this region to be non-zero. Due to the neglect of wall slip effects, discrepancies may exist between the simulated and actual velocity distributions. In practical pipeline transport, the slurry velocity may be higher than that predicted by the numerical model.

### 3.3. Distribution Characteristics of Particle in Long-Distance Pipeline Flow

**Figure 12:**
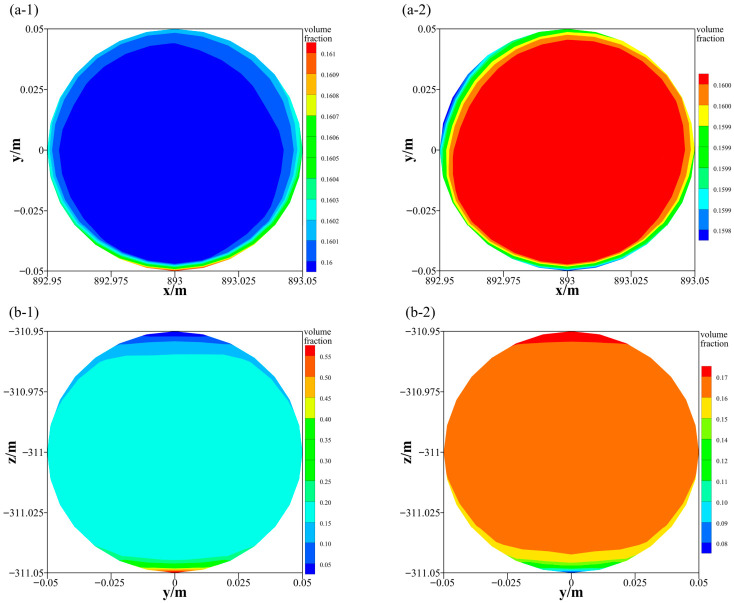
Volume fraction distribution (**a-1**) GT in vertical pipe section; (**a-2**) FT in vertical pipe section; (**b-1**) GT in horizontal pipe section; (**b-2**) FT in horizontal pipe section.

In numerical simulations, the occurrence of particle settling can be evaluated based on the volume fraction of each phase. If the volume fraction is uniformly distributed across the same cross-section, no settling or suspension occurs; otherwise, settling or suspension is indicated. To investigate the volume fraction distribution of the slurry within the pipe cross-section, the horizontal pipe section III and the vertical pipe section II were selected as representative sections for analysis. The corresponding results are presented in Figure 12. As shown, the slurry volume fraction in both horizontal and vertical sections exhibit a pronounced stratified distribution. The difference between the top and bottom regions of the pipe is particularly significant, whereas the volume fraction in the central region remains relatively uniform, with only minor or negligible variation.

**Figure 13:**
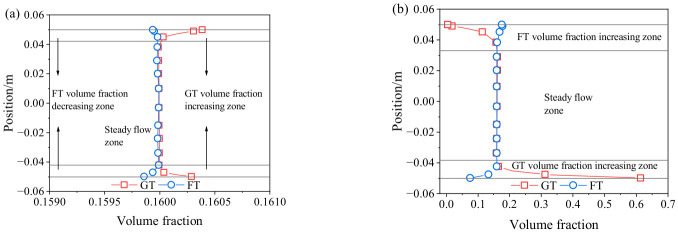
Volume fraction distribution (**a**) in vertical pipe section; (**b**) in horizontal pipe section.

The volume fraction profiles along the reference line of the monitored cross-section were extracted, and the results are presented in Figure 13. As shown in Figure 13a, the volume fraction distributions of GT and FT exhibit a concentric pattern, with both maintaining values of approximately 0.16. For GT, a relatively higher volume fraction is observed near the pipe wall, indicating a volume fraction increasing zone, whereas the volume fraction in the core zone remains relatively uniform, corresponding to a steady flow zone. In contrast, FT exhibits an opposite distribution pattern. This phenomenon can be attributed to the difference in particle size between GT and FT, which leads to variations in the forces acting on the particles. Due to its larger particle size, GT experiences greater gravitational and inertial forces, promoting its migration toward the pipe wall and consequently increasing the local volume fraction in that zone. In contrast, FT particles, being smaller in size, are less affected by gravity and inertia but are more susceptible to turbulent transport toward the pipe center, resulting in an increased volume fraction in the central zone.

As shown in Figure 13b, the volume fraction in the horizontal pipe section no longer follows a volume fraction distribution but instead exhibits a gradient variation from the bottom to the top of the pipe. For GT, the bottom region corresponds to a volume fraction increasing zone, the middle region represents a relatively steady flow zone, and the top region is characterized by a volume fraction decreasing zone. In contrast, FT exhibits an opposite distribution pattern. The volume fraction partitioning observed in this study is in good agreement with the results reported by Liu et al. [28]. This behavior is primarily attributed to particle size induced gravitational differences, which lead to particle segregation. Larger GT particles are more prone to settle at the pipe bottom under gravity, whereas smaller FT particles tend to remain suspended and migrate toward the upper zone of the pipe.

**Figure 14:**
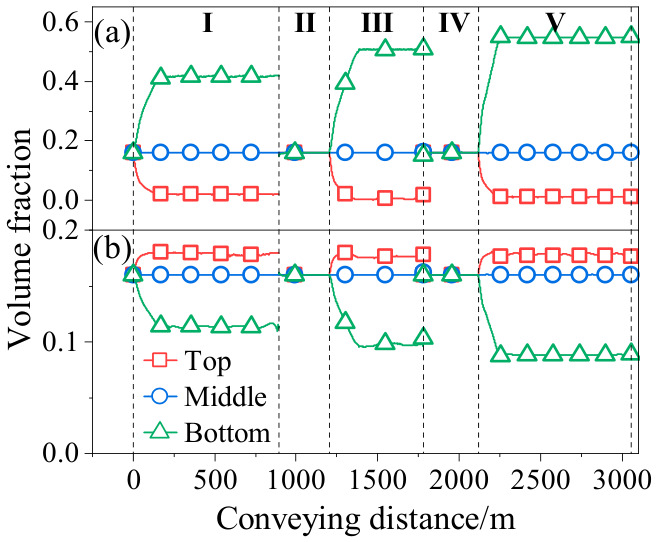
Variation in volume fraction with conveying distance (**a**) GT; (**b**) FT.

To further investigate whether the slurry exhibits zonal characteristics in volume fraction during the transport process, the top, bottom, and central axis of the pipe were selected as reference lines [28]. The volume fraction along these lines was extracted and is presented in Figure 14. Figure 14a illustrates the relationship between the volume fraction of GT and the transport distance. It can be observed that the volume fraction at the top of the pipe initially decreases with increasing transport distance and then remains relatively stable. In contrast, the trend at the bottom of the pipe is opposite, whereas the volume fraction in the core region is essentially unaffected by the transport distance and remains nearly constant throughout. For FT, as shown in Figure 14b, the distribution trend of volume fraction within the pipe is opposite to that of GT. Furthermore, for both GT and FT, the stratification of volume fraction is more pronounced in the horizontal pipe section than in the vertical section.

In practical pipeline transport, shear-induced particle migration can also influence the volume fraction distribution. However, in the present study, the effect of wall slip induced by shear-driven particle migration on the volume fraction distribution was not considered. This omission may lead to a potential misinterpretation of particle distribution in actual pipelines, such as the overestimation of volume fractions near the bottom and top regions and an underestimation in the central region. Therefore, particular attention should be paid to this issue in practical applications.

At the initial stage of transportation, intensified particle collisions and the imbalance between gravitational force and fluid drag acting on the particles lead to suspension or settling phenomena. As the conveying distance increased, particle segregation was further aggravated. When the collision forces among tailings particles reached a dynamic equilibrium with the gravitational and fluid drag forces, the influence of conveying distance on the volume fraction distribution gradually diminished and eventually became negligible.

**Figure 15:**
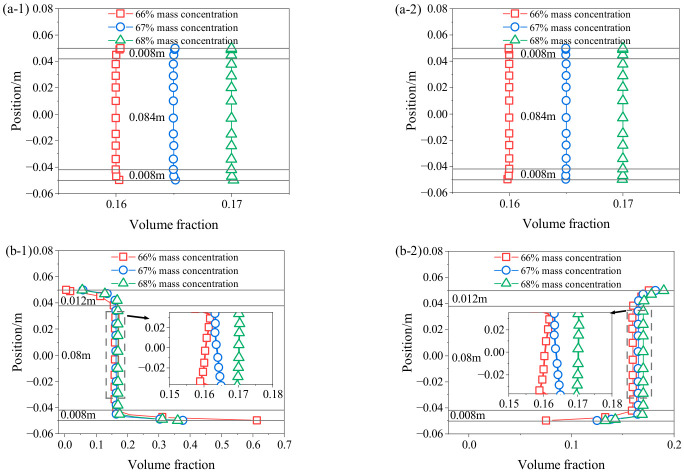
Volume fraction distribution at different mass concentrations (**a-1**) GT in vertical pipe section; (**a-2**) FT in vertical pipe section; (**b-1**) GT in horizontal pipe section; (**b-2**) FT in horizontal pipe section.

Figure 15 shows the relationship between the volume fraction and radial position of the pipeline cross-section under different mass concentrations. It can be seen from Figure 15(a-1,a-2) that the particle volume increasing or decreasing zone is 8 mm, and the steady flow zone is 84 mm. It can be observed from Figure 15(b-1,b-2) that for GT, the particle volume increasing zone is 8 mm, the steady flow zone is 80 mm, and the particle volume decreasing zone is 12 mm. For FT, the particle volume decreasing zone is 8 mm, the steady flow zone is 8 mm, and the particle volume increasing zone is 12 mm. At the same pipeline position, volume fraction increases with mass concentration, as higher concentration raises the proportion of tailings and thus increases particle volume fraction. Notably, at a mass concentration of 66%, the maximum GT volume fraction at the pipeline bottom is 0.61, much higher than that at 67% and 68%. This may be due to the decrease in slurry concentration, which increases free water, expands the spacing between solid particles, and weakens the interaction force between particles, making it easier for particles to settle and suspend.

**Figure 16:**
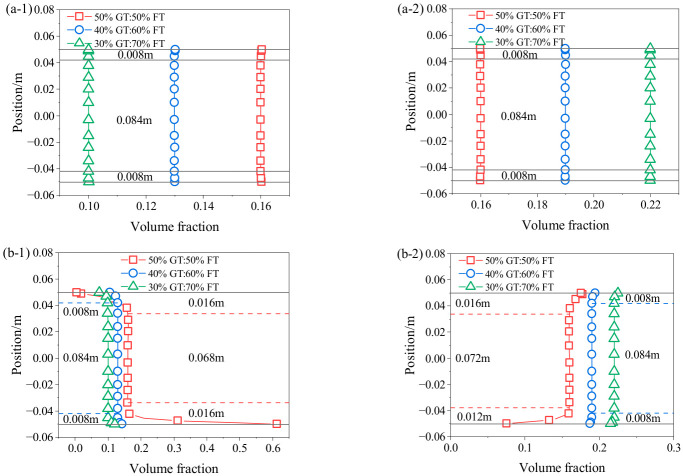
Volume fraction distribution at different particle size distributions (**a-1**) GT in vertical pipe section; (**a-2**) FT in vertical pipe section; (**b-1**) GT in horizontal pipe section; (**b-2**) FT in horizontal pipe section.

Figure 16 shows the relationship between the volume fraction and radial position of the pipeline cross-section under different particle size distribution. The results indicate that at the same position of the pipeline, the FT volume fraction increases, and the GT volume fraction decreases with the increase in FT content, which is mainly due to the change in the proportion between FT and GT. It can be seen from Figure 16(a-1,a-2) that the particle volume increasing or decreasing zone is 0.008 m, and the steady flow zone is 84 mm. It can be observed from Figure 16(b-1,b-2) that with the increase in FT content, the particle volume increasing and decreasing zone shrinks, while the steady flow zone expands. For GT, when the GT content decreases from 50% to 30%, the particle volume increasing zone shrinks from 16 mm to 8 mm, the steady flow zone expands from 68 mm to 84 mm, and the particle volume decreasing zone shrinks from 16 mm to 8 mm. For FT, when the FT content increases from 50% to 70%, the particle volume decreasing zone shrinks from 12 mm to 8 mm, the steady flow zone expands from 72 mm to 84 mm, and the particle volume increasing zone shrinks from 16 mm to 8 mm. Lower GT content reduces sedimentation at the bottom, shrinks the increasing and decreasing zone, and expands the steady flow zone. Higher FT content increases the mass of FT suspended in the middle, raises the volume fraction, expands the steady flow zone, and shrinks the increasing and decreasing zone. However, the settling of coarse particles at the bottom of the horizontal pipe increases the risk of pipeline blockage. Therefore, in practical engineering applications, it is crucial to control the particle size distribution within a reasonable range.

## 4. Conclusions

To explore the influence of particle size distribution on slurry flow characteristics under long-distance transportation conditions, rheological tests and numerical simulations were carried out, and corresponding results were obtained. The main conclusions are as follows:

(1) At the same shear rate, the shear stress increases with the increase in slurry mass concentration and FT content. The yield stress and plastic viscosity of the slurry show an increasing trend with the decreasing particle size.

(2) The transportation distance is positively correlated with the slurry transportation resistance, and the transportation resistance increases with the increase in mass concentration and FT content.

(3) During long-distance transportation, particle separation occurs in the slurry in both horizontal and vertical pipe sections, and the volume fraction tends to first increase and then remain stable with increasing transportation distance. The volume fraction of the slurry in the pipeline presents a zonal distribution: in vertical pipe sections, the FT volume fraction decreases near the pipe wall, while the GT volume fraction increases. In horizontal pipe sections, the bottom and top of the pipeline are volume fraction change zones, and the middle of the pipeline is a volume fraction steady zone. When the tailings gradation is changed from 50% GT:50% FT to 30% GT:70% FT, the steady flow zone expands, and the particle volume increasing and decreasing zones shrink.

Compared with previous studies, this work provides an integrated experimental–numerical framework to systematically evaluate the influence of particle size distribution on slurry rheology and long-distance transport behavior, with particular emphasis on the volume fraction distribution. But it should be noted that thixotropy and wall slip effects of the slurry were not evaluated in the rheological tests. In addition, the particle system was simplified when describing the mixed aggregate size distribution. In the numerical simulations, constant rheological parameters were assumed throughout the pipeline. However, in practice, the rheological properties of the slurry may vary due to thixotropy, shear history, and wall slip effects. Therefore, the present numerical model is intended for the qualitative analysis of the influence of aggregate particle size distributions on flow behavior, rather than for the direct application of the simulation results to mine practice.

Future work will focus on the following aspects:

(1) Incorporating factors such as tailings size distribution width and packing structure to establish a more comprehensive relationship between aggregate particle size distributions and rheological parameters;

(2) In numerical simulations, incorporating thixotropy, time-dependent behavior, wall slip effects, and shear history to develop a more complete model for slurry pipeline transport. Furthermore, experimental validation will be conducted to verify the reliability of the numerical simulation results.

## Figures and Tables

**Table 1:** Basic physical properties of tailings.

Item	Particle Density/kg/m^3^	Dense Density/kg/m^3^	Compactness/%	Porosity/%	*d*_10_/μm	*d*_30_/μm	*d*_50_/μm	*d*_60_/μm
GT	3054	1840	60	40	9.62	31.10	54.13	76.01
FT	3146	1270	42	58	1.40	4.03	7.38	11.20

**Table 2:** Preparation plan for fresh CPB slurry.

Case No.	Cement-Sand Ratio	Mass Concentration/%	Tailings Ratio		*MPS*/μm
			GT Content/%	FT Content/%	
1	1:5	66	50	50	30.76
2		67	50	50	30.76
3		68	50	50	30.76
4		66	40	60	26.08
5		66	30	70	21.41

**Table 3:** Fitting results of rheological parameters of slurry.

Case No.	Rheological Model	τ_0_/Pa	η_0_/Pa·s	K/Pa·s^n^	n	R^2^
1	Bingham	112.43	0.29	/	/	0.89
2		150.14	0.34			0.95
3		251.35	0.85			0.95
4		152.81	0.45			0.84
5		268.91	0.96			0.97
1	Herschel–Bulkley	137.92	/	7.80	0.42	0.77
2		194.44		20.84	0.28	0.97
3		274.64		3.20	0.77	0.73
4		152.48		0.98	0.80	0.58
5		274.39		1.03	1.01	0.65

## Data Availability

The original contributions presented in this study are included in the article. Further inquiries can be directed to the corresponding author.
